# EXTL3-interacting endometriosis-specific serum factors induce colony formation of endometrial stromal cells

**DOI:** 10.1038/s41598-019-48840-8

**Published:** 2019-08-29

**Authors:** Alar Aints, Signe Mölder, Andres Salumets

**Affiliations:** 1 Institute of Clinical Medicine, Department of Obstetrics and Gynecology, University of Tartu, Tartu, 51014 Estonia; 2 Kvintest OÜ, Tartu, 50410 Estonia; 3 Competence Centre on Health Technologies AS, Tartu, 50410 Estonia; 4 Institute of Bio- and Translational Medicine, University of Tartu, Tartu, 50411 Estonia; 5 Department of Obstetrics and Gynecology, University of Helsinki and Helsinki University Hospital, Helsinki, 00014 Finland

**Keywords:** Diagnostic markers, Adaptive immunity

## Abstract

Endometriosis is a benign chronic condition characterized by the existence of endometrial-like stroma and glandular tissue in extrauterine locations. The molecular mechanisms of its pathogenesis have not been elucidated. We have studied the role of EXTL3 (exostosin-like 3) in endometriosis and found that it is expressed in endometrial tissue as well as endometriosis lesions. We have found that serum from endometriosis patients contains a factor or factors, which interact with EXTL3 resulting in strongly increased colony formation in regenerating cell culture. We also found increased anti-EXTL3 antibodies in endometriosis patients’ sera. EXTL3 is an N-acetyl glucosamine (GlcNAc) transferase, performing a key step in heparan sulfate (HS) glucosaminoglycan synthesis. Many viruses replicate in regenerating epithelial cells and use HS as a receptor for cell entry. We measured antibody titres to viruses, which use HS as a receptor for cell entry, and found rarely increased titres for these viruses in endometriosis sera, whereas titres to viruses using other receptors were equally distributed in study groups. The data indicate that perturbation of HS metabolism is associated with endometriosis.

## Introduction

Endometriosis is defined as the existence of endometrial-like stromal and glandular tissue in extrauterine locations, most often on the peritoneum in the abdominal cavity, or in the ovaries. It is responsive to cyclic hormonal changes, thereby undergoing bleeding and regeneration, resulting in significant discomfort. It is a very common disease occurring in 6 to 10% of women^1,2^. Different sources report a slightly different association with symptoms. In women with pain, or infertility, the occurrence is 35–50%^3^. About 25 to 50% of infertile women have endometriosis, and conversely, 30 to 50% of women with endometriosis are infertile^4^.

Specific molecular mechanisms resulting in ectopic formation of endometrial tissue have not been identified. Serum factors have been found to induce umbilical cord stromal cell differentiation into endometrial epithelial cells, however, the nature of these factors, nor their target molecule has not been identified^5^. Autoimmunity has also been associated with endometriosis. Endometrial epithelium has been described as highly immunogenic, and antibody levels to endometrial surface antigens have been found to correlate to endometriosis stage^6^. To investigate endometriosis-associated autoantibodies, we have previously used phage display 7 amino acid library panning using immunoglobulin G (IgG) antibodies purified from endometriosis patients’ sera. This method identified, among others, a peptide with homology to EXTL3^7^.

EXT proteins function as N-acetyl glucosamine (GlcNAc) transferases involved in synthesis of glycosaminoglycan (GAG), specifically heparan sulfate (HS). HS synthesis proceeds initially by transfer of xylose (Xyl) to a specific serine residue in the protein. This takes place in the endoplasmatic reticulum. Further modification by two galactose (Gal) units and a glucuronic acid (GlcA) is completed in the Golgi apparatus to yield the common linker tetrasaccharide βGlcA-1,3-βGal-1,3-βGal-1,4-βXyl-(Serine). Then, HS and heparin synthesis is continued by adding a GlcNAc residue by exostosin family transferases, like EXTL3^8^.

We hypothesized, that since many viruses infect epithelial stem cells, they may be related to the pathogenesis of endometriosis. DNA viruses have been studied in this context, and contradictory results have been found^9–11^. Interestingly, HS is a cell entry receptor for many viruses, such as papilloma- and herpesviruses. Binding to and entry into cells for these viruses requires intact and correctly sulfated HS^12^. Since EXT proteins are involved in the synthesis of these viral entry receptors, we decided to compare the distribution of antibody titers to viruses, which use HS as a receptor vs. titers to viruses using different receptors.

EXTL3 also functions as a signaling receptor. In keratinocytes, it has been found to inhibit terminal differentiation and increase cell proliferation via activation of phosphatidylinositol 3 kinase (PI3K) and the kinase AKT. EXTL3 signaling is increased in psoriasis, where it is mediated by overexpression of regenerating islet-derived protein 3a (REG3A), a cognate ligand for EXTL3, as well as an antimicrobial lectin^13^. Mutations in *EXT* family genes are associated with multiple exostoses syndrome, characterized by excessive growth of bone tissue resulting in osteochondroma formation and deformities. Therefore, EXTL3 was chosen as a plausible study candidate for a target molecule in endometriosis; a disorder involving excessive cellular proliferation.

## Materials and Methods

### Patients

All patients signed an informed consent form before the donation of serum and biopsies, approved by the Research Ethics Committee of the University of Tartu (219/M-15). All methods were performed in accordance with the relevant guidelines and regulations. All patients were studied laparoscopically in Tartu University Hospital and endometriosis was confirmed or excluded. Removed endometriosis foci were kept on ice in sterile cell culture medium and processed the same day. The samples from healthy controls were collected during routine ambulatory visits. The healthy controls were not laparoscopically studied (because they had no complaints), while infertile patients were laparoscopically proven to be endometriosis-free. For statistical analysis the healthy and infertile groups were pooled and labelled as endometriosis-free (N). There was no difference between the healthy and infertile subgroups regarding the studied parameters. The first ELISA study, focused on viral titers, involved 9 endometriosis patients, 8 endometriosis-free infertile patients and 8 healthy controls. The endometriosis group included 7 stage I-II patients and two stage III patients. Four were also diagnosed with infertility. The second ELISA study, focused on autoantigenic targets, involved 15 endometriosis patients, 15 endometriosis-free infertile patients and 14 healthy controls. The endometriosis group included two stage I-II patients and 13 stage III-IV patients. The sera were collected using BD Vacutainer Clot Activating Tubes (Ref#367896), sera were aliquoted after clotting and stored at −80 °C until use. Endometrial biopsies were collected using Pipelle de Cornier Mark II, Laboratoire CCD, Paris, France, Ref#111020100.

### PCR

RNA was extracted from endometrioma capsule material with Qiagen RNA extraction kit (Qiagen, Germany) and reverse transcribed using Sigma AMV Reverse transcriptase (Sigma-Aldrich, USA). Functional EXTL3 mRNA expression was detected with primers EXTL3 5′2510: CCTCACCCAGATACCTCCGCAA and EXTL3 3′3022: CTCTCCACACCCGGAACCCAAA. Full-length cDNA was cloned with primers EXTL3 5′734 CCCCTCGAG**ACC**ATGACAGGCTATACCATGCTGC and EXTL3 3′3505 CCCGGTACC**CAA**GATGAACTTGAAGC. The forward primer contained a consensus translation initiation sequence element (ACC, bold) and reverse primer contained mutated stop codon (bold). The truncated EXTL3-ΔC construct was prepared with primer EXTL3 3′1971-S CGTGCTGGTACCGGGAACATTGCCTCCAAGC. The PCR products were cleaved with XhoI and KpnI restriction enzymes (sites underlined), ligated into pEGFP-N3 vector (Clontech, USA) and verified by sequencing.

### Cells

Primary eutopic endometrial stromal cells (ESC) were prepared from biopsies using collagenase (Sigma-Aldrich, USA) digestion for 1 hour at 37 °C. The cell suspensions were filtered through 50 µm and 35 µm strainer (Cell Strainer Cap, BD Falcon, USA) to separate cells from undigested tissue fragments. The cells were plated in DMEM/F12 medium with 10% (v/v) FBS, penicillin, streptomycin and amphotericin B and passaged twice to remove epithelial cells.

For the experiments, endometrial stromal cells from endometriosis-free patients E343 and E305 (primary cells ESC-343 and ESC-305) were plated on 6-well plates (Greiner Bio One International GmbH, Germany) and transfected the following day using FuGENE 6 transfection reagent (Promega Corporation, Madison, WI, USA) and 2 µg plasmid DNA per well. Human serum (10% v/v) and estradiol (10 µM) was added 4 hours after transfection. The cells were photographed 24 h after transfection, returned to the incubator and left unattended until colonies formed. Live cell imaging was done on a Nikon Eclipse TS100 inverted microscope equipped with Digital Sight imaging system.

### Immunostaining

The cells were washed in PBS, and fixed in 10% (w/v) formaldehyde for 15 min at room temperature. The cells were washed again, and blocked in 2% (w/v) human albumin in PBS overnight at 4 °C. Antibodies were added in blocking solution for 4 hours at room temperature. Anti-SUSD2-phycoerythrin conjugate, clone W5C5 was purchased from BioLegend, San Diego, California, USA, Cat#327406 Lot#B167847, and anti CD9-FITC conjugate was purchased from Novus Biologicals, Cambridge, UK, Cat#NB110-81617, Lot#520464. DAPI (D9542, Sigma-Aldrich) was used to stain nuclei. Microphotos were taken using Cytation 5 cell imaging multi-mode reader from BioTek, Winooski, VT, USA.

### ELISA

Peptides (Table 1) were synthesized and biotin-conjugated to 96-well ELISA plates at Jena Peptide Technologies GmbH (Jena, Germany). All wells were in duplicate. The plates were blocked in PBS-Tween-20 (0.1% v/v)-Human Albumin (0.05% w/v) overnight, incubated with serum diluted 1:100 in blocking buffer for 1 hour, washed 4 times in PBST and incubated with secondary goat-anti-human IgG-HRP (Horse Radish Peroxidase) (Labas OÜ, Tartu, Estonia) for one hour, washed 4 times, developed with SigmaFast OPD substrate (P9187-50, Sigma-Aldrich, Germany) for 5 minutes and read using Multiscan EX reader (Thermo Electron corporation, USA). The optical density (OD) readings were averaged and normalized to both blank and human IgG-coated wells using formula T = (ODexp-ODblank)/(ODIgG-ODblank) * 100. The experiments were not blinded, samples belonging to different groups were processed in parallel in identical manner.

**Table 1:** Peptides used in ELISA assays.

Name	Amino acid sequence
EXTL3-3	VTDFYRSW
PGRMC1	EDVVATGADPSDLESG
HPV16 E6	FYSKISEYRHYSYSLYGTTL
HPV155 L1	DQNPPPEKKDPYDEYTFWKV
HPV19 L1	DKNPPKEKVDPYKNLHFWNV
HSV1 gB	PANAATRTSRGYEDQGPL
CMV UL11	VQNNDYVYWSFGGGGGNRLM
VZV D	ASKYNSVGSKASLNGSGT
EchoV E11 VP1	AVPALTPGETGHTSQVTPS
HBoV VP1	QLHDHAYSELIKSGKNPYLY
HPyV7 VP1	SNQRTVQAGYGPAAVQEVT

### Statistics

For ELISA tests results, Student’s t-test p values (two-tailed) were calculated between Endometriosis (E) and Endometriosis-free (N) groups using MS Excel. The rationale for pooling healthy and infertile subgroups against E was that if the test results were to be used for diagnostic purposes, they should be able to detect endometriosis against healthy as well as unhealthy background. To correct for multiple testing, FDRq values were calculated in R 3.2.2. Graphs were produced in R using Boxplot and Beeswarm modules. All data points represent the normalized average of two technical replicate readings.

## Results

### EXTL3 expression and colony formation in culture

#### EXTL3 expression

**Figure 1:**
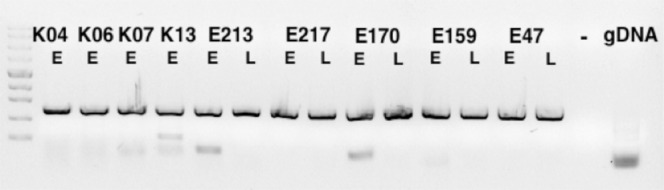
*EXTL3* PCR products of 535 bp were produced from all cDNAs from eutopic endometrium (E) and endometriosis lesions (L). K04-K13 – healthy controls, E213-E47 – endometriosis patients, “−” – no template, gDNA – genomic DNA. Marker – Fermentas ZipRuler Express II.

**Figure 2:**
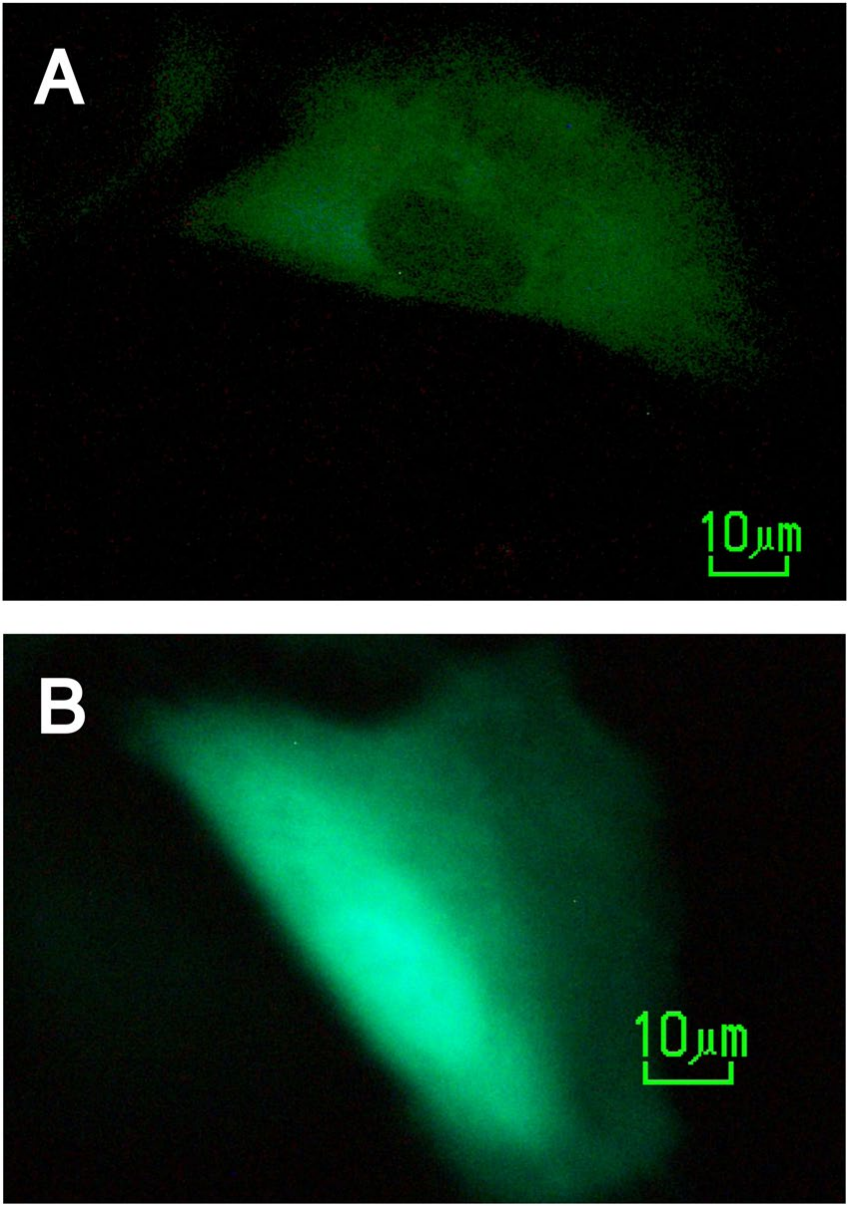
Fluorescently tagged protein expression in live transfected endometrial stromal cells (**A**) EXTL3-EGFP (**B**) control EGFP.

EXTL3 expression in endometrial tissue has not been reported previously. Multiple RNA isoforms are produced from the *EXTL3* gene in most tissues, several of which yield short cytoplasmic protein forms^14^. Therefore, PCR primers were designed targeting the exons required for full-length transmembrane protein expression. All 14 tested cDNAs from normal eutopic endometrial samples and samples from endometriosis lesions yielded a PCR product of expected 535 bp size (Fig. 1). One endometrioma from each patient was analyzed. All samples produced an easily detectable PCR band. We conclude that EXTL3 expression in endometrium is ubiquitous in patients and healthy individuals and not different in eutopic and endometriosis samples. Full length *EXTL3* was cloned from eutopic endometrial cDNA using primers providing an improved translation initiation sequence and a mutated stop codon for fusion to enhanced GFP coding sequence. The constructs were verified by sequencing. To verify the construct’s functionality, the plasmid encoding the fusion gene was transfected into primary endometrial stromal cells and EGFP signal was observed in membrane-associated pattern as expected (Fig. 2A). The transfection efficiency was estimated to be <1%.

#### Colony formation in culture

**Figure 3:**
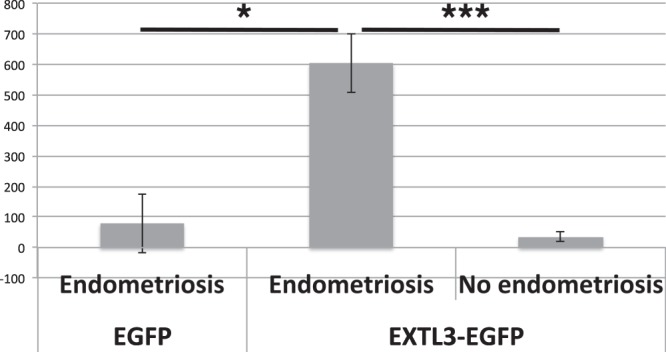
Numbers of regenerating colonies in wells transfected with EXTL3 or control vector and grown with sera from endometriosis patients or endometriosis-free patients (mean ± SD). *p = 0.002, ***p = 9.5 × 10^−5^, one-tailed t-test.

The EXTL3-transfected endometrial stromal cells from endometriosis-free patients, and the same cells transfected with control EGFP vector were grown in DMEM-F12 with antibiotics, 10% Fetal Calf Serum (FCS) (heat-inactivated), 10 µM estradiol, with 10% added human serum. The sera were not pooled. We tested 4 individual sera from endometriosis patients, and three sera from patients found negative for endometriosis by laparoscopy. EGFP signal was undetectable 4 days after transfection. The growth medium was not changed, and the cells were left unattended in the incubator for 6 weeks. The majority of the cells died, however, colonies of regeneration were observable. At 6 weeks, the number and size of the colonies was significantly different between the groups (Table 2 and Fig. 3). Significantly more colonies grew when both EXTL3 and endometriosis serum were present (EXTL3 vs control - p = 0.002, Endometriosis vs control - p = 9.5 × 10^−5^, one-tailed t-test). These results indicate that the factors present in serum are specific to endometriosis and interact with EXTL3.

**Table 2:** Numbers of *in vitro* regenerating colonies depend on EXTL3 expression and endometriosis serum.

Diagnosis	Serum ID	EGFP	EXTL3-EGFP^a^
Endometriosis	E297	21	644
	E319	26	682
	E344	223	624
	E355	44	462
Infertile, no endometriosis	E353		35
	E356		18
	E358		54

^a^On average 78 (EGFP) vs 603 (EXTL3-EGFP) colonies grew with endometriosis patients’ sera; both n = 4, t-test, one tailed p = 0.002; and 35 (infertile patient sera, n = 3) vs 603 (endometriosis patient sera, n = 4) for EXTL3-EGFP transfected cells, t-test, one tailed, p = 9.5 × 10^−5^.

**Figure 4:**
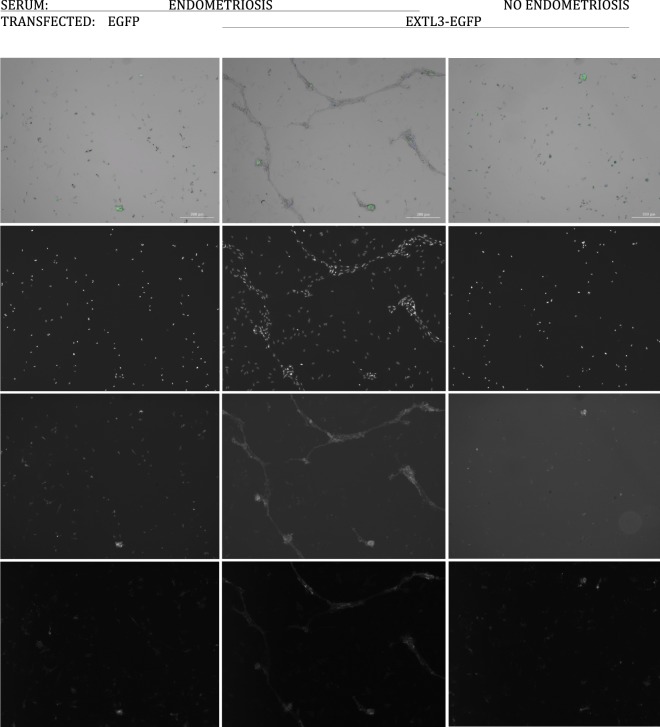
Regenerating colonies and glandular structures in culture. Column 1 – Transfected with EGFP, cultured with sera from an endometriosis patient. Column 2 – Transfected with EXTL3-EGFP, cultured with sera from an endometriosis patient. Column 3 – Transfected with EXTL3-EGFP, cultured with serum from a patient without endometriosis. Row 1 – composite image of bright field, DAPI (blue), CD9 (green), and SUSD2-W5C5 (red). The red signal is considerably weaker compared to the others. Row 2 – DAPI, row 3 – CD9, and row 4 – SUSD2 W5C5.

The colonies were EGFP negative and stained positive with W5C5 antibody (a marker for stromal stem cells) and CD9 antibody (a marker for endometrial epithelial cells). Surrounding cells around the colonies did not stain, serving as internal control (Fig. 4).

We wished to find out whether a truncation mutant of EXTL3 lacking the C-terminal glycosyltransferase domain had activity in this assay, in order to narrow down the functionally relevant domain. The *EXTL3-*Δ*C* construct coding for amino acids 1-660 of EXTL3 was prepared by PCR and expressed as fusion protein to EGFP in an identical manner to the full-length protein. In the following experiment, using eutopic endometrial stromal cells from a different donor (E305, who had infertility, but no endometriosis), the cells were transfected with wtEXTL3-EGFP, EXTL3-ΔC-EGFP, and EGFP plasmids and cultured with 5% human serum from an endometriosis patient. The colonies formed after two months, and were counted as follows: wtEXTL3 – 210; EXTL3-ΔC – 215; EGFP – 58; not transfected – 65; and NHS (no human serum) – 0. The results indicate that the serum factors interact with the N-terminal portion of EXTL3 and its glycosyltransferase domain is dispensable for colony formation activity.

### Serum antibodies

#### EXTL3 antibodies

**Figure 5:**
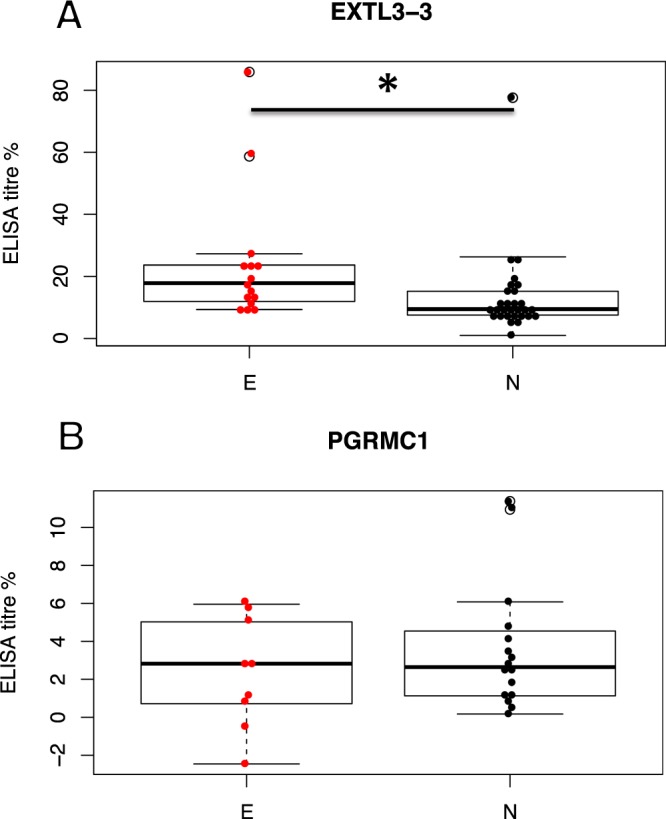
(**A**) Serum antibody titers to EXTL3, as measured by ELISA, are increased in endometriosis. * - Student’s two-tailed t-test p = 0.042, E: MEAN = 24.1; SEM = 5.42; n = 15 vs. N: MEAN = 13.21; SEM = 2.53; n = 29. (**B**) titers to PGRMC1 do not differ between groups. Student’s two-tailed t-test p = 0.37, E: MEAN = 2.39; SEM = 0.80; n = 9 vs. N: MEAN = 3.61; SEM = 0.84; n = 16. E – endometriosis, N – Endometriosis-free.

We had previously found EXTL3 antibodies in endometriosis serum. We therefore extended the search and designed synthetic peptide ELISA kits to measure the serum antibody titers to defined epitopes in target proteins. The study groups did not differ regarding age (average 33 years), height (average 166 cm), body weight (average 62 kg), and body mass index (average 22), t-test p > 0.1. We considered that the unstudied healthy control group might have occult endometriosis at the rate typical to general population (6–10%). The serological test results were above background levels in some of the control group members. These were considered as false positive findings, due to lack of confirmatory evidence of endometriosis. However, the groups were still statistically different. We found increased antibody titers to EXTL3 peptide 3 in endometriosis group (E) compared to endometriosis free (N) group (Fig. 5A). In comparison, antibodies to another endometrial cell surface antigen, progesterone receptor membrane component 1 (PGRMC1), were not significantly elevated in any of the study groups (Fig. 5B).

#### Antibodies to viruses

**Figure 6:**
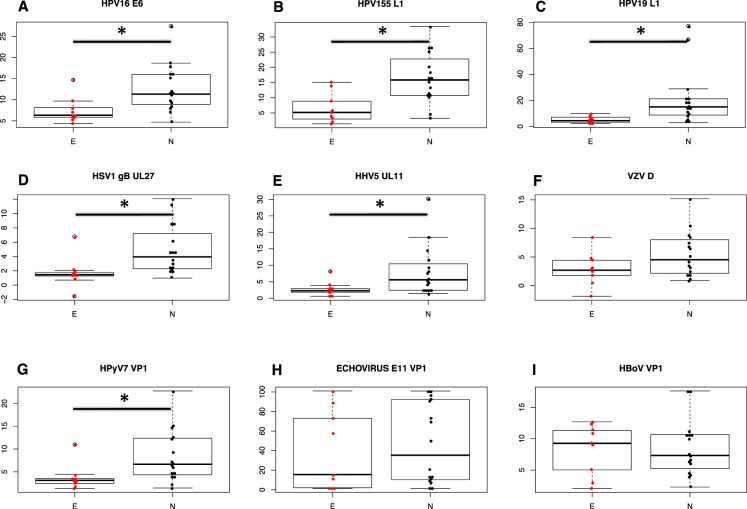
Serum antibody titers to viral antigens, as measured by ELISA, depend on the virus entry receptor and whether the patient has endometriosis. In endometriosis group (E) the titers are rarely increased against viruses binding to heparan sulfate (HPV16, HSV1, CMV), whereas in Endometriosis-free (N) group the titers are higher (* - q < 0.05). Titers against viruses binding sialic acid receptors (Echovirus and Bocavirus) are not significantly different between the groups. (**A**) HPV16-E6, p = 0.0064, FDRq = 0.0346; (**B**) HPV155-L1, p = 0.0012, FDRq = 0.0270; (**C**) HPV19-L1, p = 0.0096, FDRq = 0.0346; **(D**) HSV1, p = 0.01, FDRq = 0.0346; (**E**) CMV (HHV5), p = 0.018, FDRq = 0.0436; (**F**) VZV, p = 0.08, FDRq = 0.0685; (**G**) human polyomavirus 7 (HPyV7), p = 0.013, FDRq = 0.0388; (**H**) Echovirus VP1, p = 0.64, FDRq = 0.3697; and (**I**) Human bocavirus (HBoV) VP1, p = 0.94, FDRq = 0.4867 (Student’s two-tailed t-test, E (n = 9) vs. N (n = 16)).

Next we measured antibody titers to various viruses we thought might be involved in endometriosis. The study groups did not differ regarding age (average 32 years), height (average 167 cm), body weight (average 66 kg), and body mass index (average 24), t-test p > 0.1. We found significant differences between titers to viruses using HS for cell entry. Interestingly, in endometriosis patients the titers were lower, i.e. very seldom increased above background, compared to endometriosis-free group (N), comprising healthy and infertile subjects. This applied to papillomavirus capsid antigens (L1) as well as early intracellular antigen E6. The differences between healthy and endometriosis-free infertile subgroups were non-significant. The titers for HPV16 E6 antigen, HPV155 L1 and HPV19 L1 antigen differed significantly between the E and N groups (Fig. 6A–C). We also found significantly fewer elevated titers for HSV-1 and CMV (cytomegalovirus, HHV5), but not for VZV (varicella zoster virus) in the endometriosis group (Fig. 6D–F). The antibody titers for human polyomavirus 7 (HPyV7) are significantly less frequently increased in endometriosis group (Fig. 6G). The antibody titers for other viruses, which do not use heparan sulfate receptors, but are known to use sialic acid (SA) did not differ between the study groups. Antibodies for a human echovirus E11 peptide (89% similar to E31 and E19) were equally present in all groups (Fig. 6H), also, the antibodies for human bocavirus VP1 peptide were equally distributed (Fig. 6I).

## Discussion

Unknown serum factors from endometriosis patients have been found to induce umbilical cord mesenchymal stem cell differentiation into endometrial cells^5^. Our data confirm and extend these findings, identifying a target molecule for the serum factors as EXTL3. We found increased anti-EXTL3 antibodies in endometriosis patients’ sera. EXTL3 is a membrane signaling molecule and has been documented to signal via PI3K pathway^13^. Antibody-mediated aggregation may contribute to such signaling mode, while at the same time inhibiting glycosyltransferase function and altering BMP and FGF pathways. We cannot rule out the presence of other factors in addition to EXTL3 antibodies, or other possible targets. We have not measured the levels of REG3A, a ligand of EXTL3.

As EXT proteins, including EXTL3 perform one of the key steps in HS synthesis, we measured antibody titers to viruses, which use HS as a receptor for cell entry, and found rarely increased titers for these viruses in the endometriosis group, whereas titers to viruses using other receptors were equally distributed between study groups. This applied to viruses from different families. Viral binding to and entry into cells requires attachment to HS, while the binding is blocked by soluble HS^12,15^. Our finding that antibody titers to HS-binding viruses are seldom increased in endometriosis patients may indicate that replication of HS-requiring viruses is hampered, possibly by reduction of synthesis of HS, reduction of specific sulfation, or by soluble HS. Concurrently, we cannot exclude the possibility that the difference in antiviral antibody titres stems from reduced exposure. Endometriosis-associated dyspareunia may reduce the number of sexual contacts and, consequently, sexually transmitted viruses, such as papillomavirus and herpesvirus. Associations between endometriosis, sexual behaviour and viruses should be studied in more detail in the future. In our results, the t-test p-values ranked neatly according to the published HS specificity of the viruses (Table 3).

**Table 3:** The statistical significance values of the titer differences (t-test, two-tailed) follow the reported receptor usage of the viruses.

Virus	Receptor	t-test p	FDR q
HPV155	ND	0.0012	0.0270
HPV16	HS	0.0064	0.0346
HPV19	ND	0.0096	0.0346
HSV1	HS	0.01	0.0346
HPyV7	Not SA	0.013	0.0388
CMV	HS and FcGR	0.018	0.0436
VZV	IGF2R, MAG	0.08	0.0685
Echovirus	SA	0.64	0.3697
HBoV	SA	0.94	0.4867

ND – not determined specifically, FcGR – Immunoglobulin G receptor.

HPV16 binds HS^16^. The exact receptor specificity has not been elucidated for HPV155 and HPV19, but their tropism is similar to HPV16, i.e. skin and vagina^17^.

Among herpesviruses, the use of GAG for cell entry is variable. HSV-1 attaches by glycoprotein D (gD) to 3-O-sulfated HS on nectin and herpesvirus entry mediator (HVEM). gD is one of the very few proteins known to bind this HS modification specifically. Glycoprotein B (gB) of human herpesvirus 5 (CMV) also binds HS but also other receptors. Both HS-deficient Chinese hamster ovary cells and fibroblast cells with enzymatically removed HS had 40% reductions in gB binding, whereas removal of chondroitin sulfate had no effect, however, a significant proportion of gB is able to associate with the cell surface in the absence of HS via an undefined nonheparin component^18^. HHV3, varicella-zoster virus (VZV) on the other hand has been found to bind insulin-like growth factor 2 receptor (IGF2R, also known as cation-independent mannose-6-phosphate receptor, CI-MPR)^19^ or myelin-associated glycoprotein instead^20^, neither of which have been reported to carry sulfated GAGs.

Polyomaviruses are very common viruses on human skin, seropositivity has been reported between 35–90%^21^. The receptor for HPyV7 has not been identified, but the receptor binding site at the capsid of HPyV7 is significantly different from other polyomaviruses, whose receptor is sialic acid and HPyV7 has been shown not to bind SA^22^. It is therefore possible that HPyV7 uses HS as its receptor. In contrast, titres to viruses known to bind SA for cell entry were equally distributed. Echoviruses bind to SA residues on CD55^23^. Human bocavirus (HBoV) is a ssDNA virus, also binding to SA^24,25^.

We therefore propose that the data we have collected is indicative of HS metabolism perturbations being associated with endometriosis, perhaps being a part of its pathogenesis. HS regulate many aspects of cell biology. In multiple exostoses syndrome, caused by mutated EXT, the osteochondroma development is caused by excessive bone morphogenetic protein (BMP) signaling that would normally be regulated by HS^26^. Therefore it is possible that the serum antibodies inhibiting the EXTL3 activity or interactions reduce HS synthesis resulting in disinhibition of BMP signaling. Among the BMP family members, BMP7 induces mesenchymal-to-epithelial transition in the metanephrogenic blastema and leads to the development of nephron tubules and glomeruli^27^. Conversely, its attenuated expression leads to epithelial-to-mesenchymal transition (EMT) and fibrosis^28^. BMP7 has been shown to signal via BMPR1A^29^. BMPR1A is highly expressed in uterus^30^. BMP7 is regulated by HS. In osteoblastic cells, cell surface-attached HS is required for BMP7 signaling-induced SMAD phosphorylation, and can be blocked by heparin or heparitinase treatment^31^. The biological activity of fibroblast growth factor (FGF) family proteins is also regulated by interactions with HS, which facilitates receptor-ligand complex formation^32^. FGF family proteins are involved in mesenchymal stem cell differentiation^33–35^. Interestingly, this interaction is facilitated also by soluble, partially degraded HS. We hypothesize that the change in balance of cell-attached vs. soluble HS may change the balance of BMP and FGF signaling and contribute to endometriosis pathogenesis. Large amounts of soluble HS are released during ovulation in follicular fluid, where it functions as an anticoagulant^36^. This HS in combination with EXTL3 antibodies may induce differentiation of resident mesothelial cells^37^ or endometrial stromal cells present due to retrograde menstruation. Somatic stem cell origin of endometriosis has been proposed, however, cell-intrinsic factors, such as genetic and epigenetic differences have been focused on before^38^. As EXTL3 is a signaling cell surface receptor for REG3A, as well as a glycosyltransferase, it is capable of regulating the balance of HS-bearing receptor signaling vs. REG3A signaling. Antibodies targeting EXTL3 may block HS synthesis, or induce signaling through the PI3K, or both. The fact that colony formation required the presence of both the overexpressed EXTL3 and endometriosis serum, indicates a positive interaction. However, it is also possible that EXTL3 interacts with soluble HS, or that the antibodies target HS.

Endometriosis is strongly associated with infertility^4^. It is therefore puzzling why the factors predisposing to such an unfavorable condition have been preserved in evolution. The current finding is that endometriosis patients appear to be significantly less frequently infected with DNA viruses, particularly the oncogenic HPV16, whereas the control group seropositivity is comparable to published average healthy population levels^17^.

In conclusion, we have detected *EXTL3* expression in endometrial tissue as well as endometriosis lesions. We have found that serum from endometriosis patients contains a factor or factors, which interact with EXTL3 resulting in strongly increased colony formation in regenerating cell culture. We have found that antibody titers to viruses, which use HS as a receptor for cell entry, are rarely increased in the endometriosis group, whereas titers to viruses using SA show no difference between groups. Since EXTL3 is involved in HS synthesis, we measured antibody titers to EXTL3 and found increased titers in endometriosis group. However, the etiological factors inducing EXTL3 autoantibodies and HS metabolism perturbation still remain to be identified.

## Supplementary information

Supplementary info

## Data Availability

All data generated or analysed during this study are included in this published article (and its Supplementary Information files).
