# Supplementary material for: EXTL3-interacting endometriosis-specific serum factors induce colony formation of endometrial stromal cells
Source: Sci Rep. 2019 Aug 29;9:12562. doi: 10.1038/s41598-019-48840-8 (PMC6715673; doi:10.1038/s41598-019-48840-8)
Supplement: Supplementary file 1 — Supplementary info [file 41598_2019_48840_MOESM1_ESM.docx]

EXTL3-interacting endometriosis-specific serum factors induce colony formation of endometrial stromal cells

Alar Aints ^a,b*^, Signe Mölder ^c^ and Andres Salumets ^a,c,d,e^


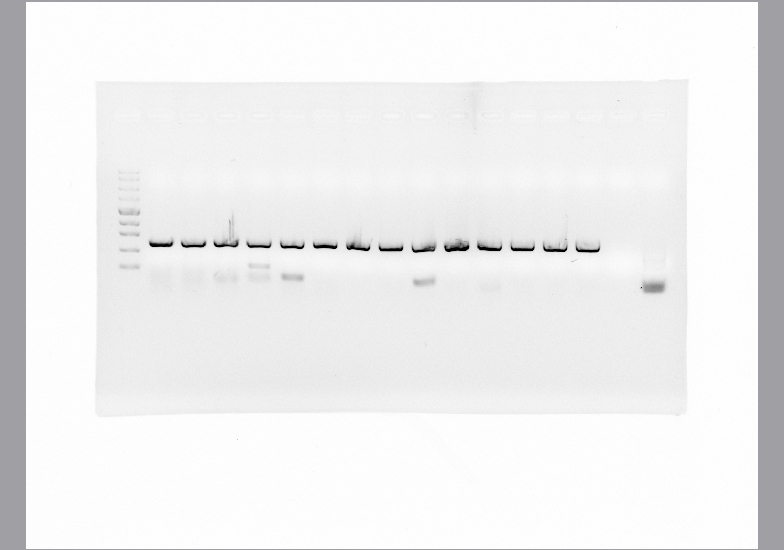


Supplementary Figure 1 – Uncropped.
